# Patient dose management in digital radiography

**DOI:** 10.2349/biij.3.2.e26

**Published:** 2007-04-01

**Authors:** E Vano, JM Fernandez Soto

**Affiliations:** Medical Physics Department, San Carlos University Hospital; and Radiology Department, Computense University, Madrid, Spain

**Keywords:** Digital radiography, patient dose, DICOM header audit, quality assurance

## Abstract

**Purpose:**

To present the experience in patient dose management and the development of an online audit tool for digital radiography.

**Materials and methods:**

Several tools have been developed to extract the information contained in the DICOM header of digital images, collect radiographic parameters, calculate patient entrance doses and other related parameters, and audit image quality.

**Results:**

The tool has been used for mammography, and includes images from over 25,000 patients, over 75,000 chest images, 100,000 computed radiography procedures and more than 1,000 interventional radiology procedures. Examples of calculation of skin dose distribution in interventional cardiology based upon information of DICOM header and the results of dosimetric parameters for cardiology procedures in 2006 are presented.

**Conclusion:**

Digital radiology has great advantages for imaging and patient dose management. Dose reports, QCONLINE systems and the MPPS DICOM service are good tools to optimise procedures and to manage patient dosimetry data. The implementation of the ongoing IEC-DICOM standard for patient dose structured reports will improve dose management in digital radiology.

## INTRODUCTION

While digital techniques in radiology have the potential to reduce patient doses, they also have the potential to significantly increase them. This is a technology that is advancing rapidly and will soon affect hundreds of millions of patients. If careful attention is not paid to the radiation protection issues of digital radiology, medical exposure of patients will increase significantly without concurrent benefit [1].

Patient dosimetry and evaluation of image quality are basic aspects of any quality control (QC) program in diagnostic radiology. Image quality must be adequate for diagnosis and obtained with reasonable patient doses. No dose limit applies to medical exposure to patients but diagnostic reference levels (DRLs) or reference values (RVs) have been proposed by the International Commission on Radiological Protection (ICRP) [2,3], and specific legislation and guidelines requiring Member States to adopt such DRLs have been published in the European Union (EU) [4,5].

The implementation of digital radiography techniques can entail an increase in patient radiation doses [1] if a strict QC program is not launched in parallel. One of the main causes for the increase is the wide dynamic range of the digital imaging systems, which allows overexposure with no adverse effect on image quality. In addition, the lack of specific training in the new digital techniques for some radiographers and the lack of well established methods to audit patient doses in digital systems can worsen the problem of patient exposure.

The ICRP became aware of this risk and launched several specific recommendations to manage patient doses in digital radiology [1]. These recommendations include appropriate training, particularly in aspects of patient dose management, revision of DRLs and frequent patient dose audits. In addition, the ICRP recommended that the industry promote tools that inform radiologists, radiographers and medical physicists about exposure parameters and the resultant patient doses.

Some EU countries require patient dose evaluation of a sample of patients of standard size for a standard procedure in all X-ray rooms on a yearly basis, as well as comparison of the results with the DRLs. If DRLs are consistently exceeded, appropriate corrective action and investigation of the causes are required to reduce doses while maintaining suitable image quality [6]. The large dynamic range of digital radiology modalities could result in patient overexposure for long periods if patient dosimetric audits are only performed on an annual basis, as typically, for conventional screen-film radiography. With conventional screen-film radiography, systematic overexposure is readily apparent because of elevated film blackening. This is not the case with digital techniques, and implementation of continuous patient dose monitoring instead of isolated annual evaluations will help to improve patient protection by avoiding systematic overexposures for long periods.

The purpose of this paper is to describe the different methods of patient dosimetry reporting and the experience with some software-assisted audit systems to survey patient doses online.

## METHODS

Practical experience and some of the presented results have been obtained in a university hospital with 965 beds and 336,840 radiological examinations in 2004. All the digital modalities of the Radiology Department send their images to a PACS, connected with a workstation of the Medical Physics Service, to extract the information contained in the DICOM header and to audit image quality with a dedicated software called “QCONLINE”. Four interventional cardiology laboratories (working with an independent PACS) have also been connected to this workstation. The transition from conventional to digital radiology started in 1999 in that hospital.

The first “online patient dose monitoring system” was described for CR auditing in a previous publication [7]. Three X-ray generators (Philips Optimus 50) were linked directly to a personal computer through the patient data organizer (PDO) system, also from Philips. The technical parameters for exposure were sent to a workstation in the Medical Physics Department, where they were kept in a database and an automatic evaluation was done based on the calculation of the varying average values of patient entrance dose (PED) and dose-area product (DAP) from the 10 most recent patients, for each examination type. Comparison of averages with DRLs gave rise to warning messages when DRLs were exceeded, prompting corrective action.

Since this initial experience, the auditing system was empowered by processing further information from the DICOM header, which currently is not restricted to only doses. Now, data on relevant exposure parameters and details on the imaging procedure are also provided. As a link with images, demographic and technical data have been implemented, allowing image quality also to be audited and to accomplish the whole QC process on an individual basis, if required, keeping dosimetric and procedural parameters related with the clinical images. The analysis of DICOM headers also permits the evaluation of modalities other than CR, such as flat detectors (DR), interventional radiology and cardiology (XA), and computed tomography (CT), depending on the contents of the corresponding header.

The three DR rooms of the Radiology Department are connected to the online audit system (General Electric Senograph 2000d for mammography, General Electric Revolution Xqi for chest and Philips Digital Diagnost for trauma examinations). The department also has eight conventional rooms and four mobile X-ray units digitised with five CRs (AGFA CR Compact and CR 75), three helical CT (General Electric HiSpeed), one multislice CT (Philips Brilliance - 64 slices) and two interventional radiology rooms (a Philips Allura FD20 flat-panel unit and a Toshiba DFP2000 unit). All these modalities are connected to an AGFA Impax 5 PACS through a fast Ethernet network. The Interventional Cardiology Department includes three Philips Integris rooms (3000 and 5000) and a Philips Allura FD that are connected to a Philips Inturis PACS. Both departments are under a quality assurance program developed by the Medical Physics Department of the hospital.

Images from each examination are sent to the corresponding PACS and then automatically routed to a workstation at the Medical Physics Department. Then the in-house software based on Microsoft Visual Basic 6.0 receives and presents the images, extracts the DICOM header and adds it to a database. At the workstation, a survey of relevant parameters (depending on the information contained in the DICOM header of each modality) is performed by comparing their current values for a given imaging procedure with values considered suitable (DRLs in the case of dosimetric data). A warning message is presented on the screen for parameters out of range, thus corrective action can be undertaken if required. By default, images received from flat panels, CR systems and interventional laboratories (only one frame per series in this case, initially) are presented on the QC workstation screen for basic image quality inspection, so that it is possible to monitor it in real time. By software, images giving rise to a warning are stored in the workstation hard disk with the alarm source recorded as another attribute in a private field at its DICOM header.

The DICOM header of each modality has been analysed to identify the fields that are useful for auditing purposes:

1. CR images contains information about examination, plate identification and number of uses, exposure level (parameter defined by the manufacturer), and processing parameters. Audited parameters are the number of exposures in the plate and exposure level. No information is provided about technique that can be used to calculate patient doses, so for this modality the first prototype of the online audit system is still in use, in additional to DICOM header analysis. It is based on a direct connection between the X-ray generator and a computed, rather than registered, technical parameters (kVp, mAs, focus, distance focus-film and collimators position) after each exposure. These data are sent to a workstation in the Medical Physics Department where the software calculates the entrance surface dose by using the X-ray tube output, which is measured periodically as part of the QC program. (PED is the absorbed dose in air at the surface of the patient in the centre of the irradiated area, including the backscattered radiation from the patient). For each examination type, a standard patient thickness is assumed for entrance dose estimation. The computer application also allows online comparison of the mean patient dose value for a recent sample with the local diagnostic reference levels in order to audit dose levels and introduce corrective action if necessary.
2. Mammography DR images contain all the technique parameters (kVp, mAs, focus size, distance focus-detector, anode and filter selection, manual or automatic exposure mode, compression force, compressor position, patient thickness, detector temperature, etc.), and a calculation of PED and glandular dose. Most of these parameters are audited and the dose calculations are verified periodically by using the results of the QC programme.
3. Chest and trauma DR images also contain all the technique parameters and a calculation of PED and DAP that are audited and periodically verified.
4. Interventional radiology and cardiology modalities were acquired with a DAP meter, which depending on the manufacturer, include this value in the DICOM header. Other useful information for audit purposes are number of frames per series, runs per procedure, kVp, mA, pulse time, distances and C-arm angulations.
5. Recent CT units include in its header information, CTDIvol, kVp and mA that can be used in auditory.

The last development in the system (still a work in progress) is a new module to collect and process the relevant information transferred by the MPPS DICOM service, that could be specially useful for XA and CT modalities because it provides information regarding the whole study such as total fluoroscopy time or total PDA (and cumulative air kerma for some of the new systems) in XA procedures, and dose length product (DLP) in CT.

Most of the interventional radiology systems have at present, the capability to produce “patient dose reports” containing relevant information to help in the audit process and to detect abnormal dose values, which are very useful in the optimisation process. Total DAP for fluoroscopy and image acquisition, total fluoroscopy time, and radiographic techniques for the different series (including sometimes the DAP per series) are reported.

The International Electrotechnical Commission (IEC) is working on a standard (recognised as “DICOM-DOSE”) written in concert with DICOM WG-02. In the standard, it is proposed that an “irradiation object” be stored for each irradiation event, irrespective of the storage of the images produced by that irradiation. The irradiation objects, along with other information, shall be stored in a “Radiation Dose Structured Report” (RDSR). The RDSR could be archived in the RIS or PACS or perhaps transferred to a “Radiation Safety Reporting System” (RSRS).

The IEC “new work item proposal”, identified as 62B/645/NP, and proposed by Germany on 12 January 2007 was circulated for voting until 20 April 2007, with the name “Radiation dose documentation – Part 1: Equipment for radiography and radioscopy”. The scope of the document encompasses all forms of projection radiographic equipment incorporating the means for measuring or calculating dose-related quantities, and capable of producing DICOM compatible images and/or reports. The document provides specific units and quantities. It does not apply for dental radiography and radioscopy, mammography and computed tomography.

## RESULTS

Results are reported for projection radiography as PED is equivalent to entrance air kerma (with backscatter). DAP values shall be understood as equivalent to air kerma area product.

At the beginning of the CR system implementation and during the transition from conventional screen-film to digital radiology in the centre studied, mean PED values were 30% higher in certain rooms, as compared with those found in conventional screen-film radiography rooms. This was mainly due to lack of training of the radiographers with regard to the new systems, especially in rooms without automatic exposure control, since the image quality after post-processing was poor only in cases of underexposure.

The QCONLINE system has been in service for more than 3 years. During this time, a significant part of the procedures carried out at the hospital has been audited as a pilot action. For mammography, images from over 25,000 patients, over 75,000 chest images, 100,000 CR procedures and more than 1,000 interventional radiology (IR) procedures, have been used.

Owing to the QA running programme, very few alarm signals were generated on mean values out of range. For chest examinations, for example, only three cases of mean values above 0.3 mGy for PA projection were observed during the initial 18 month period. For IR, alarms were mainly related to procedures exceeding 2,500 frames (in cardiology). Local reference values (RVs) (calculated as the 3^rd^ quartile of the dose distributions) resulted between 30% and 60% lower than the entrance surface dose (ESD) of European reported RVs, while showing good image quality (as reported by the radiologists in charge of these evaluations).

Some EU countries’ regulation on quality assurance programmes requires patient dose evaluation of a sample of patients of standard size for a standard procedure in all X-ray rooms on a yearly basis, and this system gives the possibility to evaluate patient doses in all of them instead of in a sample, thus any deviation can be immediately corrected.

**Figure 1:**
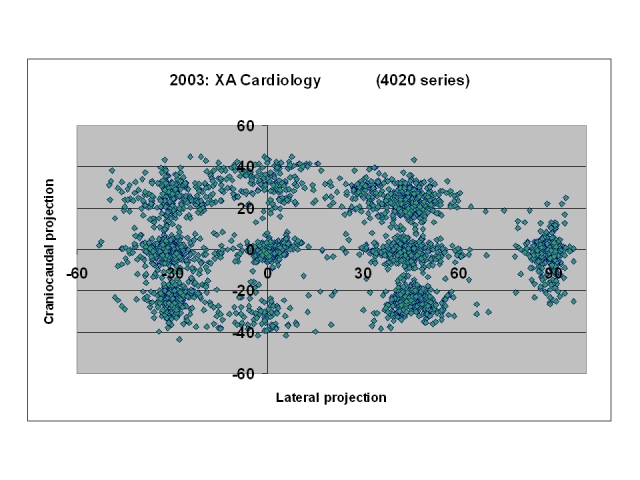
Common projections in a cardiology room obtained from the DICOM header.

Another important exploitation of the DICOM header information contained in the cine series of the cardiology procedures is the orientation of the X ray beam. Figure 1 presents a graph of this orientation for a sample of 4,020 series. This information allows estimation of the level of scatter dose in the cardiology laboratories (very dependent on the C-arm orientation).

**Figure 2:**
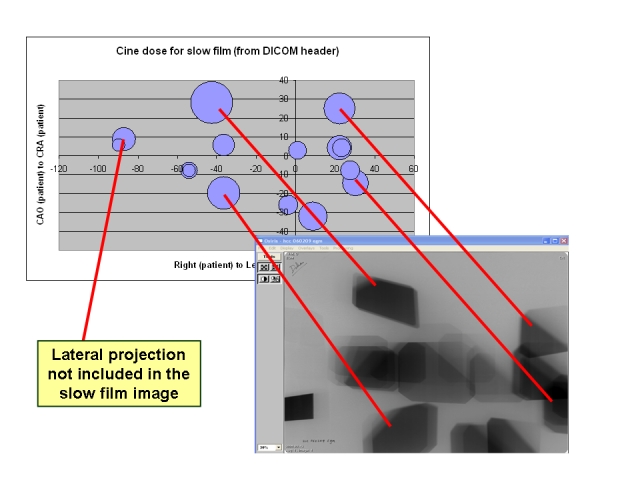
Use of DICOM header information to calculate skin dose distribution.

The information on the angulations of the C-arm, together with the radiographic technique of the different series (or the DAP or cumulative dose per series) and the geometrical data (distances and radiation field size) allows calculation of the skin dose distribution. Figure 2 presents one example of this calculation, which is compared with the experimental skin dose distribution measured with a slow film position between the table and the patient.

**Figure 3:**
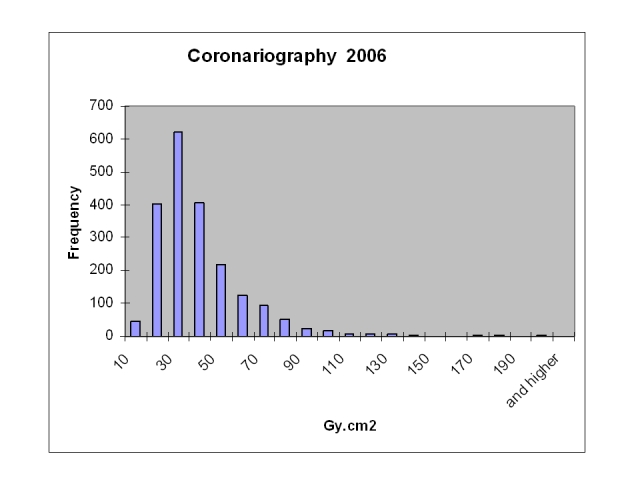
Patient dose distribution for coronary angiography.

**Figure 4:**
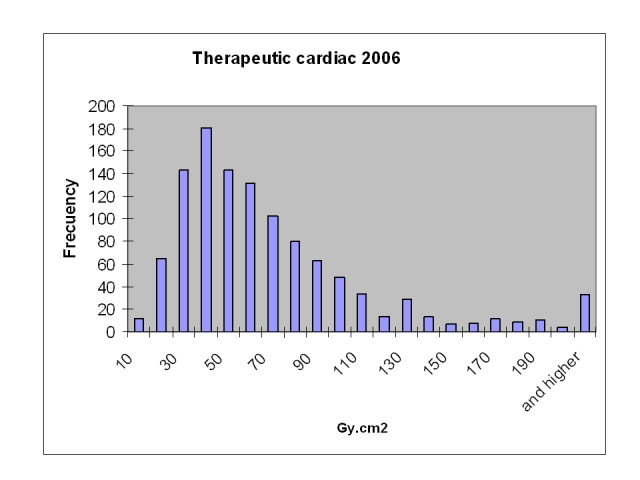
Patient dose distribution for coronary therapeutic procedures.

The transfer of the patient dose reports to a database allows the collection of large samples for statistical calculations of patient doses and to have access to individual dose data in the case of repeated procedures or high individual dose data. Table 1 and Figures 3 and 4 show the results for the cardiology procedures during 2006 at the centre.

**Table 1:** Statistical results of the cardiology procedures during 2006.

**Year 2006**	**Coro (Gy.cm²)**	**PTCA (Gy.cm²)**	**Coro + PTCA (Gy.cm²)**
Mean	34.9	72.6	60.7
Median	29.2	53.4	51.9
Stand. dev	22.0	66.4	41.4
Min	1.3	5.4	5.1
Max	198.2	676.4	474.5
Sample	2038.0	544.0	599.0

## DISCUSSION AND CONCLUSION

Systems of QCONLINE have demonstrated their benefits to manage patient doses in digital radiology. The pilot system described operated long enough to establish its reliability and has demonstrated the possibility offered by the contents of the image DICOM header to monitor dose levels in real time, to compare them with DRLs and later to analyse the causes producing abnormal values, based on inspection of other chosen parameters such as exposure mode and technical parameter set. The possibility of implementing this QC system with a direct link to the modalities, without the need of a PACS, is another interesting feature. The aim is to implement it in small centres, on whatever system including the DICOM storage services, and through the intranet real-time dose monitoring system that has been described as an appropriate quality control tool to ensure that radiation doses remain within selected norms. Because of the ease with which doses at CR can be increased, eluding identification of the problem for an extended period, this type of tool allows replacement of advantageous annual dosimetric evaluations in patients.

## ACKNOWLEDGMENTS

Supported in part by the European Commission (SENTINEL coordination action FP6-012909), the Spanish Ministry for Science and Technology (project FIS2006-08186), and the Spanish Nuclear Safety Council.
